# Management and outcomes of women with low fibrinogen concentration during pregnancy or immediately postpartum: A UK national population‐based cohort study

**DOI:** 10.1111/aogs.14828

**Published:** 2024-03-22

**Authors:** Caroline Diguisto, Elfreda Baker, Simon Stanworth, Peter W. Collins, Rachel E. Collis, Marian Knight

**Affiliations:** ^1^ National Perinatal Epidemiology Unit, Nuffield Department of Population Health University of Oxford Oxford UK; ^2^ Pôle de Gynécologie Obstétrique, Médecine Fœtale, Médecine et Biologie de la Reproduction, center Olympe de Gouges, CHRU de Tours Université de Tours Tours France; ^3^ NHS Blood and Transplant Oxford UK; ^4^ Oxford University Hospitals NHS Trust Oxford UK; ^5^ Institute of Infection and Immunity Cardiff University Cardiff UK; ^6^ Department of Anaesthetics Cardiff and Vale University Health Board Cardiff UK

**Keywords:** fibrinogen, hemorrhage, placenta, postpartum, pregnancy

## Abstract

**Introduction:**

Pregnant women with a fibrinogen level <2 g/L represent a high‐risk group that is associated with severe postpartum hemorrhage and other complications. Women who would qualify for fibrinogen therapy are not yet identified.

**Material and methods:**

A population‐based cross‐sectional study was conducted using the UK Obstetric Surveillance System between November 2017 and October 2018 in any UK hospital with a consultant‐led maternity unit. Any woman pregnant or immediately postpartum with a fibrinogen <2 g/L was included. Our aims were to determine the incidence of fibrinogen <2 g/L in pregnancy, and to describe its causes, management and outcomes.

**Results:**

Over the study period 124 women with fibrinogen <2 g/L were identified (1.7 per 10 000 maternities; 95% confidence interval 1.4–2.0 per 10 000 maternities). Less than 5% of cases of low fibrinogen were due to preexisting inherited dysfibrinogenemia or hypofibrinogenemia. Sixty percent of cases were due to postpartum hemorrhage caused by placental abruption, atony, or trauma. Amniotic fluid embolism and placental causes other than abruption (previa, accreta, retention) were associated with the highest estimated blood loss (median 4400 mL) and lowest levels of fibrinogen. Mortality was high with two maternal deaths due to massive postpartum hemorrhage, 27 stillbirths, and two neonatal deaths.

**Conclusions:**

Fibrinogen <2 g/L often, but not exclusively, affected women with postpartum hemorrhage due to placental abruption, atony, or trauma. Other more rare and catastrophic obstetrical events such as amniotic fluid embolism and placenta accreta also led to low levels of fibrinogen. Maternal and perinatal mortality was extremely high in our cohort.

## Graphical abstract

Fibrinogen <2 g/L often, but not exclusively, affected women with postpartum hemorrhage. The catastrophic events leading to low fibrinogen are associated with extremely high maternal and perinatal mortality.

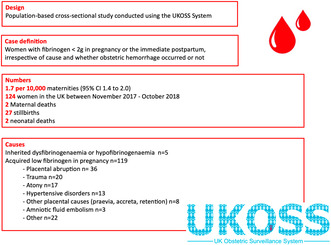

**Abbreviations**
AFE: amniotic fluid embolism
PPH: postpartum hemorrhage
UKOSS: UK Obstetric Surveillance System

**Key message**

Fibrinogen <2 g/L is a rare condition occurring in settings of severe obstetrical events that are associated with high maternal, fetal, and neonatal mortality.

## 1 INTRODUCTION

Obstetric hemorrhage is an important cause of maternal morbidity and mortality.^1^ Major obstetric hemorrhage may be associated with early coagulopathy, for example at the time of placental abruption or amniotic fluid embolism (AFE) or may evolve as dilution impacts resuscitation, further exacerbating blood loss with serious clinical implications. Fibrinogen plays a crucial role in the formation of a fibrin/platelet clot and arresting bleeding. Low fibrinogen is usually the first hemostatic indicator of coagulopathy following postpartum hemorrhage (PPH).^2^ Guidelines for the management of PPH recommend that resuscitation, in addition to all the other measures, should target a maternal plasma fibrinogen concentration ≥2 g/L during ongoing PPH.^3^, ^4^ This recommendation is based on a double‐blind randomized controlled study showing that when fibrinogen is >2 g/L, fibrinogen replacement does not affect bleeding^5^ and the findings from observational data suggesting that women with a plasma fibrinogen <2 g/L and continuing bleeding represent a high‐risk group that is associated with both progressive hemorrhage and the increased use of blood products and invasive procedures.^2^, ^6^, ^7^ However, the use of this cut‐off in algorithms for the management of PPH is difficult because (1) laboratory coagulation results are often too slow to be useful clinically and (2) there is no definitive evidence that shows that replacement of fibrinogen improves outcomes in ongoing PPH, although an observation study showed that improved outcomes were associated with early targeted fibrinogen replacement.^5^, ^8^, ^9^, ^10^ Further investigations need to clarify if early fibrinogen replacement could improve outcomes in women with PPH.^11^ To identify which women could qualify for fibrinogen therapy, the incidence of clinically significant low fibrinogen in pregnancy, defined as <2 g/L, as well as the causal pathways leading to low fibrinogen need to be determined. Indeed, previous studies have shown that coagulopathy patterns observed in cases of obstetric hemorrhage depend on the underlying cause of hemorrhage, so indications of fibrinogen may also vary according to these.^12^

The aims of this study were to estimate the incidence of low fibrinogen in pregnancy in the UK, and to describe the characteristics of women and the probable causes of this fibrinogen deficiency alongside their management and outcomes.

## 2 MATERIAL AND METHODS

### 2.1 Research design and setting

A population‐based study was designed using the UK Obstetric Surveillance System (UKOSS), a research platform that collects national population‐based information on specific severe pregnancy complications. All 194 consultant‐led maternity units in the UK participate in UKOSS; the administration is set within the National Perinatal Epidemiology Unit at the University of Oxford.

### 2.2 Case identification

For the purposes of this study, data on women with either a laboratory Clauss fibrinogen <2 g/L or one of the two point‐of‐care rapidly available hemostasis tests rotational thromboelastometry (ROTEM, Werfen, Barcelona, Spain) FIBTEM reading less than 10 mm (A5, A10, or MCF) or Thromboelastography (Haemonectics, Boston, MA, USA) reading less than 200 mg/dL at any time during pregnancy or the immediate postpartum period, i.e before discharge from hospital, were collected, irrespective of cause and whether obstetric hemorrhage occurred or not.

### 2.3 Sample size

Results from a previous UKOSS study on massive transfusion (eight or more units of red blood cells) among women with obstetric hemorrhage reported an incidence of massive transfusion of 23 per 100 000 maternities with women who had a median plasma fibrinogen at presentation of 1.9 g/L (interquartile range [IQR] 1.2–2.8).^12^ Given the number of maternities, we expected that the number of women with low fibrinogen due to PPH would be approximately 130 in a year in the UK. Considering that low fibrinogen may also occur outside PPH, the number of women with plasma fibrinogen <2 g/L within the UK obstetric population was estimated at 180 per year.

### 2.4 Data collection and consent

A nominated UKOSS obstetrician, midwife, anesthetist, or risk management coordinator from each unit transmitted monthly the number of cases or confirmed zero cases over a 12‐month period, from November 1, 2017 until October 31, 2018. Every case was given a unique anonymized UKOSS identification number. Information was entered on data collection forms and securely transferred from hospitals to the central UKOSS database, which is hosted on secure servers at the University of Oxford. Individual consent was not required for collection of anonymous data.

Collected information included women's demographic details, previous obstetric and medical history, and information on the ongoing pregnancy. The causes of low fibrinogen were defined as placental abruption, trauma (vaginal cervical laceration, uterine extension at cesarean section, uterine rupture or inversion), uterine atony, placental causes other than abruption (placenta previa, placenta accreta, retention of the placenta), AFE, hypertensive disorders (preeclampsia, HELLP syndrome [hemolysis elevated liver enzymes low platelets], liver failure, or eclampsia), early pregnancy complications (ectopic pregnancy, miscarriage, or termination of pregnancy before 14 weeks), infection, inherited dysfibrinogenemia or hypofibrinogenemia, stillbirth, or other. Hematological laboratory tests were collected including level of fibrinogen (at first and worst), hemoglobin, platelet counts, activated prothrombin time, prothrombin time (PT), and International Normalized Ratio (INR) at worst. The estimated blood loss, any management of low fibrinogen or PPH including transfusion of any blood component (red blood cells, fresh frozen plasma, or platelets) or any of cryoprecipitate, fibrinogen concentrate or tranexamic acid were reported. The need for any of the following surgery or obstetrical procedures was also collected: hysterectomy or other surgery (laparotomy and primary repair, brace suture, intra‐abdominal packing, or artery ligation), uterine tamponade or artery embolization (non‐exclusive procedures). Finally, outcomes for mothers and infants were collected.

### 2.5 Statistical analyses

The incidence rate of fibrinogen <2 g/L in pregnancy (number of reported women with low fibrinogen in pregnancy or immediate postpartum/number of women giving birth over the same time period across the UK) and its 95% confidence interval were estimated. Denominator data were obtained from routine statistics published by the Office for National Statistics (For England and Wales), National Records Scotland, and the Northern Ireland Statistics and Research Agency and the national Diabetes in Pregnancy Audit.

The women were grouped and separated by the primary cause of low fibrinogen, as identified by reporters. Women with more than one reported cause were allocated a primary cause based on the opinion of reporters, the order in which etiologies occurred, and the comparative severity of these problems.

All statistical analysis was undertaken using STATA SE v13.1 (StataCorp, College Station, TX, USA) with data being summarized as frequencies and percentages of the total cohort, and as medians and IQR for continuous data, due to the non‐normal distribution of variables. Missing values were excluded from these results.

## 3 RESULTS

Over the study period, 161 cases of women with fibrinogen <2 g/L were notified, with data being provided for 129 women. Five women were excluded after return of the report forms; one was a duplicated form, and four women did not fit the case definition with one delivery outside the duration period and three women not having low fibrinogen as defined in this study, leaving an overall total of 124 women. The estimated incidence rate was of 1.7 per 10 000 maternities in the UK (95% confidence interval 1.4–2.0 per 10 000 maternities).

**FIGURE 1:**
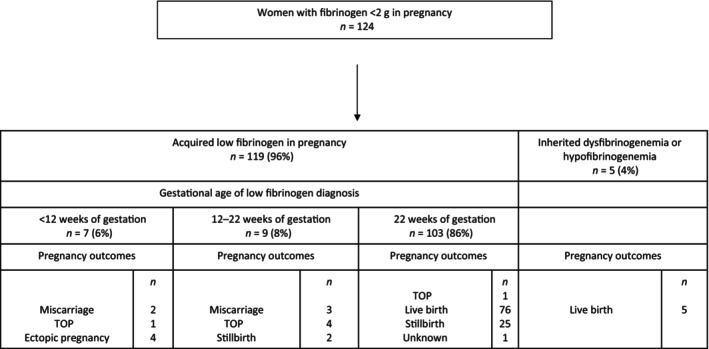
Flow chart of the population. TOP, termination of pregnancy.

The cohort included five women (4%) with inherited dysfibrinogenemia or hypofibrinogenemia (Figure 1). For these five women the low fibrinogen was diagnosed either before pregnancy or in the first trimester of pregnancy. They all had live births. Among the 119 women with acquired hypofibrinogenemia, low fibrinogen was diagnosed in the prepartum or the postpartum period and occurred mostly (86% of cases; 103/119) after 22 weeks of gestation. Seven women, including four with ruptured ectopic pregnancies, two with miscarriages, and one with a termination of pregnancy complicated by hemorrhage, had a low fibrinogen before 12 weeks of gestation. For the other nine women, low fibrinogen was diagnosed between the 12th and the 22nd week of gestation (three cases of miscarriage, four cases of termination of pregnancies and two cases of stillbirth). Thirty‐seven percent (*n* = 46/124) of women were 35 years old or older and 16% (*n* = 20/124) and 11% (*n* = 14/124) were of Asian/Asian British and Black/Black British ethnicity, respectively (Table 1). Eight percent of women (10/124) had a total estimated blood loss below 500 mL (Table 1).

**TABLE 1:** Demographic characteristics, medical history, and characteristics of ongoing pregnancy of the 124 women identified as having low fibrinogen, according to whether or not hemorrhage occurred.

	Women without hemorrhage^a^	Women with hemorrhage & pregnancy ending <22 weeks of gestation	Women with hemorrhage & pregnancy ending ≥22 weeks of gestations
	*N* = 10, *n* (%)	*N* = 19, *n* (%)	*N* = 95, *n* (%)
Demographic characteristics			
Age (years)			
<25	3 (30)	3 (16)	12 (13)
25–34	7 (70)	6 (31)	47 (49)
≥35	0 (0)	10 (53)	36 (38)
Missing	0	0	0
White ethnicity	7 (78)	10 (56)	58 (64)
Asian/Asian British	2 (22)	4 (22)	14 (15)
Black/Black British	0 (0)	4 (22)	10 (11)
Chinese/Chinese British	0 (0)	0	9 (10)
Missing	1	1	4
Medical history			
Body mass index (kg/m^2^)			
<25	4 (50)	5 (42)	45 (49)
25–30	3 (37)	4 (33)	27 (29)
≥30	1 (13)	3 (25)	20 (22)
Missing	2	7	3
History of bleeding disorders	3 (30)	3 (16)	1 (1)
Missing	0	0	0
Thrombocytopenia	0 (0)	0 (0)	2 (2)
Missing	0	0	1
Nulliparas	3 (30)	3 (16)	33 (35)
Missing	0	0	0
No previous cesarean^b^	5 (71)	10 (63)	38 (61)
One previous cesarean	2 (29)	5 (31)	16 (26)
Two or more previous cesarean	0	1 (6)	8 (13)
Missing	0	0	0
Previous postpartum hemorrhage^b^			
Yes	2 (28)	1 (69)	11 (18)
Missing	0	0	2
Characteristics of ongoing pregnancy			
Multiple pregnancy^c^	1 (10)	0	7 (7)
Missing	0	0	0
Antenatal anticoagulant prescription			
Aspirin	1 (10)	0 (0)	14 (15)
Low‐molecular‐weight heparin	0 (0)	0 (0)	8 (8)
Other	0 (0)	0 (0)	1 (1)
Missing	0	0	0
Mode of delivery^c^			
Prelabor cesarean	3 (38)	NA	45 (48)
Cesarean during labor	0 (0)	NA	14 (15)
Spontaneous vaginal delivery	5 (62)	NA	23 (25)
Instrumental vaginal delivery	0 (0)	NA	11 (12)
Missing	2	NA	2

Abbreviation: NA, non‐applicable.

^a^ Hemorrhage defined as 500 mL.

^b^ Percentage out of women with previous pregnancies over 24 weeks.

^c^ Among women with a delivery after 22 weeks of gestation.

Fifty‐seven women (46%) had multiple etiologies for the low level of fibrinogen, according to reporters. Each of those cases was assessed so that one primary cause was identified. The most frequent causes of acquired low fibrinogen were placental abruption (29%; 36/124), trauma (16%; 20/124), atony (14%; 17/124), and other placental causes (6%; 8/124) (Table 2). Three women had low fibrinogen due to causes of hemorrhage that were not related to pregnancy. There were no clear causes for the observed low fibrinogen for three women, with one woman experiencing a minor PPH and two women not experiencing any significant bleed. These women had broadly good outcomes, and their low fibrinogen did not seem to be symptomatic of any problem, or indicative of severe results.

**TABLE 2:** Estimated blood loss and biological findings presented according to the primary cause of low fibrinogen.

	Estimated total blood loss (L)^a^	Fibrinogen, g/L			Hb, g/dL at worst^a^	Platelets 10^9^/L < 75 000^b^	APTT (s) at worst^a^	PT (s)^a^	INR > 1.5^b^
		First^a^	Worst^a^	<0.5^b^ ^,^ ^c^					
Placental abruption (*n* = 36)	*n* = 35	*n* = 33	*n* = 30	*n* = 36	*n* = 35	*n* = 35	*n* = 31	*n* = 32	*n* = 27
	2.2 (1.5–3.5)	0.9 (0.7–1.9)	0.8 (0.6–1.7)	6 (17)	74 (68–81)	16 (46)	29 (26–39)	13 (12–16)	9 (33)
Trauma^d^ (*n* = 20)	*n* = 19	*n* = 19	*n* = 19	*n* = 20	*n* = 20	*n* = 20	*n* = 17	*n* = 19	*n* = 14
	2.2 (1.7–3.0)	1.5 (1.0–1.7)	1.3 (0.9–1.7)	2 (10)	69 (57–75)	5 (25)	28 (24–38)	13 (11–16)	3 (21)
Uterine atony (*n* = 17)	*n* = 17	*n* = 16	*n* = 16	*n* = 16	*n* = 17	*n* = 17	*n* = 15	*n* = 16	*n* = 12
	3.0 (2.8–4.2)	1.6 (1.2–1.9)	1.5 (1.1–1.8)	1 (6)	72 (59–79)	6 (35)	32 (26–39)	14 (11–16)	2 (17)
Placental abnormalities^e^ (*n* = 8)	*n* = 8	*n* = 8	*n* = 7	*n* = 8	*n* = 8	*n* = 8	*n* = 4	*n* = 7	*n* = 6
	4.4 (2.6–7.5)	1.6 (1.4–1.9)	1.4 (0.8–1.7)	0 (0)	75 (62–93)	4 (50)	29 (26–41)	13 (12–17)	0 (0)
Amniotic fluid embolism (*n* = 3)	*n* = 3	*n* = 3	*n* = 2	*n* = 3	*n* = 3	*n* = 3	*n* = 2	*n* = 3	*n* = 2
	4.4 (4.0–7.0)	0.7 (0.5–1.3)	0.2 (0.2–0.3)	2 (67)	64 (60–74)	3 (100)	46 (42–50)	18 (14–29)	1 (50)
Hypertensive disorders of pregnancy (*n* = 10)	*n* = 12	*n* = 13	*n* = 13	*n* = 13	*n* = 13	*n* = 13	*n* = 13	*n* = 13	*n* = 8
	0.7 (0.5–1.0)	1.5 (0.8–1.8)	1.2 (0.6–1.6)	3 (23)	89 (78–102)	4 (31)	32 (24–39)	13 (11–15)	1 (13)
Early pregnancy problems (*n* = 6)	*n* = 6	*n* = 5	*n* = 5	*n* = 5	*n* = 5	*n* = 5	*n* = 3	*n* = 5	*n* = 5
	1.7 (0.8–3.0)	1.4 (1.3–1.7)	1.4 (1.3–1.7)	0 (0)	73 (66–78)	0 (0)	27 (25–30)	17 (16–19)	0 (0)
Infection (*n* = 7)	*n* = 6	*n* = 7	*n* = 7	*n* = 7	*n* = 7	*n* = 7	*n* = 6	*n* = 7	*n* = 6
	0.9 (0.5–2.0)	1.3 (0.8–1.9)	1.3 (0.5–1.9)	1 (14)	87 (77–91)	1 (14)	32 (28–36)	16 (13–18)	2 (33)
Inherited dysfibrinogenemia or hypofibrinogenemia (*n* = 5)	*n* = 2	*n* = 3	*n* = 5	*n* = 5	*n* = 4	*n* = 5	*n* = 3	*n* = 4	*n* = 2
	0.8 (0.3–1.7)	0.9 (0.8–1.5)	0.9 (0.7–1.5)	1 (20)	103 (87–12.8)	0 (0)	26 (25–30)	16 (14–23)	0 (0)
Stillbirth (*n* = 3)	*n* = 3	*n* = 2	*n* = 2	*n* = 2	*n* = 3	*n* = 2	*n* = 2	*n* = 2	*n* = 2
	0.7 (0–1.4)	1.4 (1.1–1.7)	1.4 (1.1–1.7)	0 (0)	105 (60–132)	0 (0)	48 (47–49)	12 (11–13)	0 (0)
Other (*n* = 3)	*n* = 2	*n* = 3	*n* = 3	*n* = 3	*n* = 3	*n* = 3	*n* = 3	*n* = 3	*n* = 2
	4.1 (1.7–6.5)	1.5 (1.2–1.9)	1.6 (1.1–1.9)	0 (0)	65 (65–83)	0 (0)	27 (27–37)	15 (14–17)	0 (0)
None (*n* = 2)	*n* = 2	*n* = 2	*n* = 2	*n* = 2	*n* = 2	*n* = 2	*n* = 2	*n* = 2	*n* = 2
	0.7 (0.3–1.1)	1.4 (0.9–1.9)	0.9/1.8	0 (0)	102 (91–113)	0 (0)	25 (21–29)	11 (10–12)	0 (0)
Missing (*n* = 1)	*n* = 1	*n* = 1	*n* = 1	*n* = 1	*n* = 1	*n* = 1	*n* = 0	*n* = 1	*n* = 1
	0.2 (NA)	1.4 (NA)	1.4	0 (0)	89 (NA)	0 (0)		11 (11–11)	0 (0)

Abbreviations: APTT, activated prothrombin time; Hb, hemoglobin; INR, International Normalized Ratio; PT, prothrombin time.

^a^ Median (interquartile range).

^b^ *n* (%).

^c^ Either first or worst fibrinogen <0.5.

^d^ Uterine extension at cesarean section, vaginal cervical laceration, uterine rupture, or inversion.

^e^ Placenta previa, placenta accreta, manual removal of placenta.

The level of fibrinogen was the lowest among women with AFE with a median of 0.2 g/L (IQR 0.2–0.3), followed by women with placental abruption and those with inherited dysfibrinogenemia or hypofibrinogenemia, who had a median fibrinogen level of 0.8 g/L (IQR 0.6–1.7 g/L) and 0.9 g/L (IQR 0.7–1.5 g/L), respectively (Table 2).

Ten percent of women (12/124) underwent a hysterectomy (Table 3). Seventy‐five percent (93/124) of women received packed red cells, and 54% (67/124) were administered fresh frozen plasma. Surgery or obstetric procedures, blood components, and fluid and other treatment according to the primary cause are presented in Table 3 and Appendix S1. Cryoprecipitate and fibrinogen concentrate were administered to 57/123 (46%) and 16/123 (13%) women, respectively, with a total of 59% of women who received concentrate fibrinogen replacement therapy. Thirteen women had a fibrinogen level <0.5 g/L, of which only three (23%) received cryoprecipitate or fibrinogen concentrate. Seventy percent (85/124) and 30% (34/124) of women were admitted to high dependency and intensive care units, respectively.

**TABLE 3:** Numbers and percentage of surgery or obstetric procedures, blood components, and fluid and other treatment by primary cause.

	Surgery or obstetric procedures				Blood components and fluids				Other treatment		
	Hystere‐ctomy	Other surgery^a^	Balloon tamponnade	Artery embolization	Packed red cells	Fresh Frozen Plasma	Platelets	Crystalloids/Colloids	Cryoprecipitate	Fibrinogen concentrate	Tranexamic acid
Placental abruption (*n* = 36)	1 (3)	9 (25)	10 (28)	1 (3)	4 (11)	22 (61)	18 (50)	34 (94)	22 (61)	5 (14)	29 (81)
Trauma^b^ (*n* = 20)	3 (15)	3 (15)	1 (5)	0 (0)	17 (85)	10 (50)	6 (30)	17 (85)	7 (35)	2 (10)	15 (75)
Uterine atony (*n* = 17)	2 (12)	3 (18)	4 (24)	2 (12)	15 (89)	11 (65)	6 (35)	17 (100)	12 (71)	2 (12)	17 (100)
Placental cause^c^ (*n* = 8)	4 (50)	3 (38)	4 (50)	4 (50)	8 (100)	5 (63)	3 (38)	8 (100)	4 (50)	2 (25)	8 (100)
Amniotic fluid embolism (*n* = 3)	1 (33)	1 (33)	2 (67)	0 (0)	3 (100)	3 (100)	3 (100)	3 (100)	2 (67)	1 (33)	3 (100)
Hypertensive disorders of pregnancy (*n* = 13)	1 (8)	1 (8)	1 (8)	0 (0)	4 (31)	6 (46)	2 (15)	9 (70)	5 (38)	1 (8)	7 (54)
Early pregnancy problems (*n* = 6)	0 (0)	1 (17)	0 (0)	0 (0)	6 (100)	3 (50)	0 (0)	6 (100)	0 (0)	1 (17)	6 (100)
Infection (*n* = 7)	0 (0)	1 (14)	1 (14)	0 (0)	3 (43)	4 (57)	2 (29)	6 (86)	2 (29)	0 (0)	3 (43)
Inherited dysfibrinogenemia or hypofibrinogenemia (*n* = 5)	0 (0)	0 (0)	0 (0)	1 (20)	1 (20)	1 (20)	0 (0)	2 (40)	1 (20)	1 (20)	1 (20)
Stillbirth (*n* = 3)	0 (0)	0 (0)	0 (0)	0 (0)	1 (33)	1 (33)	0 (0)	1 (33)	0 (0)	1 (33)	1 (33)
Other (*n* = 3)	0 (0)	1 (33)	0 (0)	0 (0)	3 (100)	1 (33)	1 (33)	2 (67)	2 (67)	0 (0)	3 (100)
None (*n* = 2)	0 (0)	0 (0)	0 (0)	0 (0)	0 (0)	0 (0)	0 (0)	1 (50)	0 (0)	0 (0)	0 (0)

^a^ Laparotomy and primary repair, brace suture, intra‐abdominal packing, or artery ligation.

^b^ Uterine extension at cesarean section, vaginal cervical laceration, uterine rupture, or inversion.

^c^ Placenta previa, placenta accreta, retention of placenta.

Two maternal deaths occurred. Both were due to massive PPH >5000 mL (Table 4). One followed a placental abruption in the early third trimester and one was due to an AFE at term, their worst fibrinogen levels were 0.7 and 0.2 g/L, respectively. The proportion of stillbirths was 24% (*n* = 25) when considering the women with acquired low fibrinogen and births at 22 weeks of gestation or later. Two stillbirths occurred before 22 weeks of gestation. Among those stillbirths, only three were identified as the primary cause of low fibrinogen, other cases were secondary to placental abruption or another complication. Among 89 live births, 49% (*n* = 44) of children were admitted to intensive care and 2% died (*n* = 2).

**TABLE 4:** Maternal and neonatal outcomes.

	*N* = 124, *n* (%)
Maternal outcomes	
Maternal death (% of total)	2 (2)
Women who survived and were admitted to level 3 critical care (% of *n* = 117)	33 (28)
Median stay duration in days (interquartile range) (*n* = 32)	2 (1–3)
Women who survived and were admitted to level 2 critical care (% of *n* = 122)	84 (69)
Women who survived and required organ support (% of *n* = 121)	19 (16)
Infant outcomes	
Stillborn (% for *n* = 103 births ≥22 weeks of gestation for women with acquired low fibrinogen)	25 (24)
Preterm (% for *n* = 81 live births)	33 (41)
5‐minute Apgar score <7 (% for *n* = 81)	7
Admission to neonatal unit (% for *n* = 81)	39 (48)
Neonatal death (% for *n* = 81)	2 (2)

## 4 DISCUSSION

The UK incidence rate of low fibrinogen in pregnancy was 1.7 per 10 000 maternities. Fibrinogen <2 g/L often, but not exclusively, affected women with PPH due to placental abruption, atony, or trauma. Other more rare and catastrophic obstetrical events such as AFE also lead to low levels of fibrinogen. AFE was associated with the highest estimated blood loss and the lowest levels of fibrinogen.

The main strength of this study lies in its prospective population‐based design enabling confirmation from the previous studies that women with fibrinogen <2 g/L are a high‐risk group. However, despite its prospective population‐based design, the calculated incidence is lower than what was previously reported, for example, a recent single center UK‐based study on PPH reported 11 cases from 11 279 maternities (9.8/10 000).^13^ We suspect that some cases may have been missed because of the lack of testing or reporting and it is likely that our population represents only the most severe cases of women with low fibrinogen, which could explain such a high mortality rate. Unfortunately, data were missing for 32/161 (20%) women who were notified to have low fibrinogen despite the reminders that were sent to reporters. We were unable to compare the characteristics of those women with those of the cohort to rule out selection bias.

According to our study, having fibrinogen <2 g/L is a rare condition occurring in settings of severe obstetrical events that are associated with high maternal, fetal, and neonatal mortality. Trials assessing fibrinogen replacement for the management of PPH have not shown clinical improvements and this may be explained in part by intervention thresholds that were too high and an inability to recruit the most severe cases who might have benefitted from such therapy. Indeed the numbers of women with fibrinogen <2 g/L in those trials are low. In the studies of Ducloy‐Bouthors and Collins the recruitment threshold was a fibrinogen of about 3 g/L, identified using viscoelastometric testing, explaining the relatively low number of women with fibrinogen <2 g/L included.^5^, ^8^ In the study of Wikkelso et al. recruitment was based on volume of bleeding and the low incidence of a fibrinogen <2 g/L reported here is consistent with their report of very few cases of fibrinogen <2 g/L in their study.^9^It is likely that women who might benefit the most from fibrinogen therapy were not included because of the recruitment criteria. This inability to recruit women representing severe cases supports the importance of observational studies for such rare and severe conditions.

High maternal morbidity was expected but maternal mortality was exceptionally high. The proportion of mothers over the age of 35 years and those of Asian/Asian British or Black/Black British ethnicity were overrepresented in our sample. In 2018–2019, only 22% of mothers were over 35 years old in the general population (NHS Maternity Statistics), compared with 37% in this study. Similarly, mothers of Asian/Asian British ethnicity and Black/Black British ethnicity were overrepresented (16% and 11% in our population vs. 11% and 4.5% in the general population). An increased maternal age and ethnic minorities are known to be risk factors for maternal mortality, emphasizing the high risk of the population.^14^ Fetal and neonatal mortality were also unexpectedly high.^15^ Similar findings were recently reported in another observational study of women with PPH and acute obstetric coagulopathy in which fetal or neonatal death occurred in 50% of cases.^13^ Poor fetal and neonatal outcomes could be explained by placental abruption being the most frequent primary cause and the high proportion of preterm births among live births.

Biological patterns and the total blood loss varied according to the cause of low fibrinogen. Placental abruption was the commonest cause of low fibrinogen within this group, being the primary cause in 36 women, and a reported contributory cause in an additional 3 women. AFE occurred as a cause of low fibrinogen in far fewer women but may also be significant in that these women had exceptionally low levels of fibrinogen and exceptionally severe hemorrhage. Subgroups of women per cause of low fibrinogen were too small to conduct comparisons but the observed differences confirm the impression that the development of coagulopathies in obstetric hemorrhages seems to be dependent on the primary cause of bleeding, with uterine atony and trauma not showing such a strong association with developing early coagulopathies but on occasion causing significant postpartum hemorrhage and subsequent coagulopathy, as seen in this study.^16^ Conversely, both placental abruption and AFE are associated with hyperfibrinolytic disseminated intravascular coagulopathy associated with reduced fibrinogen levels and inhibition of fibrinogen function (also called acute obstetric coagulopathy^13^), alongside other obstetric causes like preeclampsia and acute fatty liver of pregnancy.^16^ Red blood cells were transfused in 75% of cases, reflecting the high volume of bleeding in many cases. Despite all the women in the study having critically low fibrinogen levels, only 59% received fibrinogen replacement in the form of cryoprecipitate or fibrinogen concentrate. Fresh frozen plasma was infused in 54% of cases; however, this product does not contain high levels of fibrinogen and so would not correct the low fibrinogen as well as cryoprecipitate or fibrinogen concentrate.^17^ It is not possible to ascertain from the data collected why relatively few women were given fibrinogen, however, UK guidelines recommend fresh frozen plasma replacement as first‐line treatment during PPH with cryoprecipitate only given for very severe bleeding and this on top of culture, the lack of evidence, and the cost may have affected clinical decision‐making.^3^

Further randomized studies are needed to investigate the efficiency of fibrinogen therapy in the management of hemorrhage with an emphasis on recruiting women with fibrinogen <2 g/L. They will be challenging as they will need take into account that indications for fibrinogen therapy may depend on the cause of low fibrinogen and hemorrhage. They will also be challenging because they involve recruiting women in critical situations, including placental abruption, AFE, and at the time of fetal demise. Finally recruiting women with low fibrinogen may be particularly difficult as it is a rare condition and because laboratory results are often too slow to be useful clinically, especially in situations of PPH. However, this could be partly compensated for by the recent development of point‐of‐care viscoelastic coagulation monitoring.^18^

## 5 CONCLUSION

Fibrinogen <2 g/L often, but not exclusively, affected women with PPH. The catastrophic events leading to low fibrinogen are associated with extremely high maternal and perinatal mortality.

## AUTHOR CONTRIBUTIONS

Marian Knight, Simon Stanworth, Peter W. Collins, and Rachel E. Collis designed the study and obtained the funding. Caroline Diguisto, Elfreda Baker, and Marian Knight conducted the analysis. Caroline Diguisto and Elfreda Baker wrote a draft of the manuscript. Caroline Diguisto, Simon Stanworth, Peter W. Collins, Rachel E. Collis, and Marian Knight revised the manuscript. All authors approved the final version of the manuscript.

## FUNDING INFORMATION

This project was funded by the National Institute for Health and Care Research (NIHR) Policy Research Program grant number PR‐PRU‐1217‐21202. Marian Knight is an NIHR Senior Investigator. The views expressed are those of the author(s) and not necessarily those of the National Health Service, the NIHR, or the Department of Health and Social Care.

## CONFLICT OF INTEREST STATEMENT

Peter W Collins has received research support from CSL Behring, Werfen, and Haemonectics, and paid consultancy from CSL Behring, Werfen, and Haemonectics. The other authors do not have any conflicts of interest.

## ETHICS STATEMENT

This UKOSS methodology received the approval of the London Multicentre Research Ethics Committee (study reference 04/MRE02/45) and this specific study received the approval from the London Brent REC1 on April 1, 2016 (REC Ref. Number: 10/H0717/20).

## Supporting information


Appendix S1.
