# Supplementary material for: Management and outcomes of women with low fibrinogen concentration during pregnancy or immediately postpartum: A UK national population‐based cohort study
Source: Acta Obstet Gynecol Scand. 2024 Mar 22;103(7):1339–47. doi: 10.1111/aogs.14828 (PMC11168278; doi:10.1111/aogs.14828)
Supplement: Supplementary file 1 — Appendix S1. [file AOGS-103-1339-s001.docx]

|  | Packed red cells^1^ | Fresh Frozen Plasma^1^ | Crystalloids/Colloids^1^  L |  |
| --- | --- | --- | --- | --- |
| Placenta abruption (n=36) | 4 [2-7] | 2[0-4] | 2.5 [2.0-3.7] |  |
| Trauma^#^ (n=20) | 3 [1-5] | 1[0-2] | 2.2 [1.7-3.7] |  |
| Uterine atony (n=17) | 4 [2-6] | 3[0-4] | 3.0 [2.5-5.0] |  |
| Placental cause^##^ (n=8) | 5 [3-17] | 4[0-8] | 2.2 [1.5-3.0] |  |
| Amniotic fluid embolism(n=3) | 7 [4-18] | 4[2-21] | 2.0 [2.0-3.7] |  |
| Hypertensive disorders of pregnancy (n=13) | 0 [0-2] | 0 [0-4] | 1.8[0.0-3.0] |  |
| Early pregnancy problems (n=6) | 3 [2-4] | 1 [1-4] | 3.0[2.0-5.0] |  |
| Infection (n=7) | 0 [0-4] | 2[0-3] | 3.7[3.0-6.0] |  |
| Inherited dysfibrinogenaemia or hypofibrinogenaemia (n=5) | 0 [0-0] | 0 [0-0] | 0.0[0.0-2.0] |  |
| Stillbirth (n=3) | 0 [0-4] | 0 [0-2] | 0.0[0.0-1.5] |  |
| Other (n=3) | 2 [2-13] | 0 [0-8] | 4.0[0.0-4.5] |  |
| None (n=2) | 0 [0-0] | 0 [0-0] | 1.2[0.0-2.5] |  |
| ^1^ Median (IQR)  ^#^ Uterine extension at caesarean section, vaginal cervical laceration, uterine rupture or inversion  ^##^ Placenta praevia, placenta accreta, retention of placenta | | | | |

**Appendix S1:** **Volume of blood components and fluids received by women**
